# Larsucosterol: endogenous epigenetic regulator for treating chronic and acute liver diseases

**DOI:** 10.1152/ajpendo.00406.2023

**Published:** 2024-02-21

**Authors:** Yaping Wang, Jenna Ren, Shunlin Ren

**Affiliations:** ^1^Department of Internal Medicine, Virginia Commonwealth University, Richmond, Virginia, United States; ^2^McGuire Veterans Affairs Medical Center, Richmond, Virginia, United States; ^3^Department of Pharmacology, Virginia Commonwealth University, Richmond, Virginia, United States

**Keywords:** Larsucosterol, DUR-928, Oxysterol sulfation, Alcoholic hepatitis, MASLD

## Abstract

Larsucosterol, a potent endogenous epigenetic regulator, has been reported to play a significant role in lipid metabolism, inflammatory responses, and cell survival. The administration of larsucosterol has demonstrated a reduction in lipid accumulation within hepatocytes and the attenuation of inflammatory responses induced by lipopolysaccharide (LPS) and TNFα in macrophages, alleviating LPS- and acetaminophen (ATMP)-induced multiple organ injury, and decreasing mortalities in animal models. Results from *phase 1* and *2* clinical trials have shown that larsucosterol has potential as a biomedicine for the treatment of acute and chronic liver diseases. Recent evidence suggests that larsucosterol is a promising candidate for treating alcohol-associated hepatitis with positive results from a *phase 2a* clinical trial, and for metabolic dysfunction-associated steatohepatitis (MASH) from a *phase 1b* clinical trial. In this review, we present a culmination of our recent research efforts spanning two decades. We summarize the discovery, physiological and pharmacological mechanisms, and clinical applications of larsucosterol. Furthermore, we elucidate the pathophysiological pathways of metabolic dysfunction-associated steatotic liver diseases (MASLD), metabolic dysfunction-associated steatohepatitis (MASH), and acute liver injuries. A central focus of the review is the exploration of the therapeutic potential of larsucosterol in treating life-threatening conditions, including acetaminophen overdose, endotoxin shock, MASLD, MASH, hepatectomy, and alcoholic hepatitis.

## Graphical abstract

**Figure:**
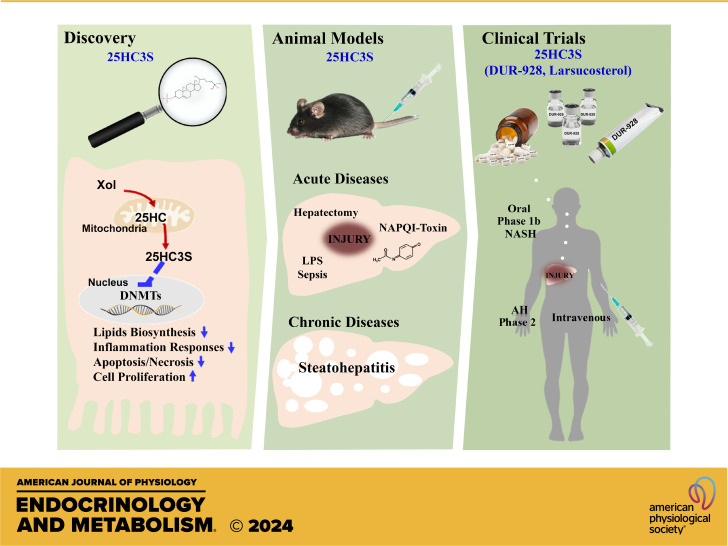

## INTRODUCTION

Epigenetics is one of the most rapidly expanding fields in biology (1). Increasing evidence has shown that epigenetic machinery is involved in the development and recovery of chronic and acute liver diseases (2–4). Thus, the use of epigenetic regulators for the treatment of diseases has become a priority in biomedical research. Epigenomic modification, especially DNA methylation, plays a key role in the regulation of gene expression (5). DNA methyltransferases (DNMTs) catalyze the reaction of DNA methylation by addition of methyl groups to DNA (6). Methylation predominantly occurs at C5 cytosine (^5m^C), specifically in proximity to guanine within CpG dinucleotides. This process is primarily concentrated in the promoter region of genes and tends to occur at a higher frequency. Hypermethylation of CpG islands is associated with gene inactivation or silencing, whereas CpG hypomethylation promotes gene activation. Global DNA hypomethylation influences genome stability (7). It was reported that DNA hypermethylation is proposed in the progression of metabolic dysfunction-associated steatotic liver disease (MASLD) in fibrotic clinical phases (8). Comprehensively, an investigation into genome-wide methylation revealed significant alterations in DNA methylation across more than a hundred genes associated with glucose and lipid metabolism, DNA damage and repair, liver tissue remodeling, and fibrosis. Transcriptional silencing of the mitochondrial gene NADH dehydrogenase 6 (MT-ND6) results from hypermethylation of its promoter and exhibits a robust correlation with the severity of MASLD (9). Thus, potent epigenetic regulators of the promoter demethylation are ideal molecules for treatment of liver diseases, such as MASLD.

There are two pathways for cholesterol degradation, bile acid synthesis: the “classic” (neutral) pathway and “alternative”(acidic) pathway (10). The classic pathway is initiated by cholesterol 7α-hydroxylase (CYP7A1) located in the microsome, whereas the alternative pathway is thought to be limited by mitochondrial sterol 27-hydroxylase (CYP27A1) (11). Mitochondria generated oxysterols, including 25-hydroxycholesterol (25HC), 27-hydroxycholesterol (27HC), and cholestenoic acid (CA), which have been shown as potent regulatory molecules (12, 13). These oxysterols play important roles in maintaining cholesterol and lipid homeostasis by regulating numerous genes associated with cholesterol and lipid metabolism as well as transportation (14–16). Interestingly, mitochondrial cholesterol uptake limits the regulatory oxysterol synthesis via the alternative pathway, which can be overcome by overexpression of the intracellular cholesterol transporter StarD1. Overexpression of StarD1 also upregulates key enzymes mediating cholesterol oxidation (Cyp7A1), biliary cholesterol secretion (ABC-G5/8), and lipid metabolism (17–20). The search for the possible mechanism of these regulatory effects led to the discovery of a novel sulfated oxysterol, 25HC3S, with potent regulatory properties (21). Assays using mitochondria isolated from rats and Cyp27A1 gene knockout mice indicated the 25HC is synthesized by Cyp27A1 and sulfated to 25HC3S by sulfotransferases 2b1 (21). Subsequently, other oxysterol sulfates have been reported to be present in human bodies (22). Recent reports have shown that 25HC, 27HC, and their sulfated forms (25HC3S, 27HC3S) are potent endogenous epigenetic regulators (23–25). 25HC/25HC3S are the most potent regulators and are dominant in hepatocyte nuclei (26). 25HC was reported as an activator of DNMT1, whereas 25HC3S was reported as an inhibitor of DNMTs (24). These paired oxysterols coordinately regulate lipid metabolism, inflammation responses, and cell survival.

DURECT Corporation (a pharmaceutical company) has named 25HC3S as DUR928, and World Health Organization (WHO) has named it as larsucosterol (27). Notably, larsucosterol administration has been reported to reduce lipid biosynthesis, attenuate inflammatory responses, and block cell death, making it a promising candidate for treating various diseases, including fatty liver diseases, diabetes, hyperlipidemia, acetaminophen (ATMP) overdose-induced liver injury, and lipopolysaccharide (LPS)-induced multiple organ injury (28–40). In the present review, we provide insight into the pathophysiological mechanisms underlying the occurrence and development of MASLD, metabolic dysfunction-associated steatohepatitis (MASH), and acute liver injury. We highlight the potential of larsucosterol as a biomedicine for the treatment of chronic and acute liver diseases, including MASLD, MASH, endotoxin shock, ATMP overdose, and alcoholic-associated hepatitis (AH).

## POSSIBLE MECHANISM OF MASLD DEVELOPMENT

MASLD, also known as nonalcoholic fatty liver disease (NAFLD), affects over 30% of the global population, making it the most common chronic liver disease worldwide (41). Despite its prevalence, there is no FDA-approved drug for its treatment (42). MASLD is characterized by abnormal lipid accumulation in liver cells, leading to inflammation, fibrosis, and severe liver-related conditions (43). Several mechanisms for MASLD development have been proposed. Two decades ago, the “two-hit” theory suggested that the initial hit involves fat accumulation and insulin resistance (44). A second hit, characterized by oxidative stress, inflammation, hepatocyte apoptosis, and activation of hepatic stellate cells, leads to the development of MASH (45). More recently, a different theory suggests that MASH and pure fatty liver are separate conditions caused by insulin resistance, with steatosis in MASH being a consequence rather than a cause of inflammation and fibrosis. The multiple hits model proposes that insulin resistance is the first hit, making the liver vulnerable to subsequent injuries over time, ultimately progressing from pure steatosis to MASH (46).

Recent research has uncovered the role of epigenomic modifications, particularly DNA methylation, in MASLD development (23). In a high glucose (HG)-cultured hepatocytes, an MASLD in vitro model, HG leads to an increase in nuclear 25HC, which specifically activates DNMT1 and regulates gene expression involved in intracellular lipid metabolism. HG significantly increases ^5m^CpG levels in at least 2,225 genes involved in 57 signaling pathways, including those related to carbohydrate and lipid metabolism such as phosphoinositide-3-kinase (PI3K), cyclic adenosine monophosphate (cAMP), insulin, insulin secretion, diabetic, and MASLD signaling pathways.

## POSSIBLE MECHANISM OF AH DEVELOPMENT

Globally, approximately two billion people consume alcoholic beverages, making alcohol abuse a leading cause of liver-associated morbidity and mortality (47). Nearly all alcohol consumers develop steatosis (48), a stage considered less injurious than advanced liver disease but strongly linked to the development of later stages of alcoholic liver disease (ALD) (49). This condition results from alcohol’s toxic effects on hepatic lipid metabolism, progressing from simple hepatic steatosis to alcoholic steatohepatitis, fibrosis, and cirrhosis. The severity of hepatic steatosis is highly associated with the development of later stages of ALD (50). Alcohol-induced hepatic lipid metabolism involves altered hepatic lipid uptake, de novo lipid synthesis, fatty acid oxidation, hepatic lipid export, and lipid droplet formation and catabolism, leading to hepatic lipid accumulation (51). The global dysregulation of lipid metabolism indicates that epigenetic regulation, specifically CpG methylation in promoter regions causing chromatin condensation and gene silencing, plays a crucial role in the development, although the detailed mechanism has yet to be explored. To date, more than 20 studies have been conducted to identify alcohol-related DNA methylation signatures (52). Most of these studies focused on alcohol dependence in relation to “global” methylation levels or preselected candidate genes, with only a few using epigenome-wide approaches. Limited sample sizes, however, have hindered the search for a robust alcohol-related DNA methylation signature.

**Figure 1.**
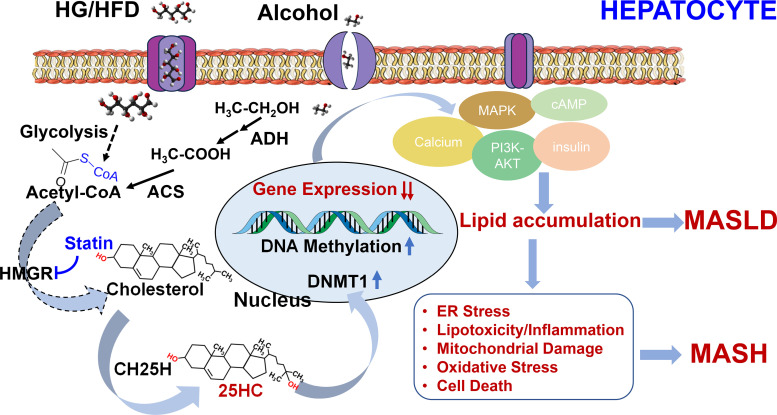
A novel pathophysiological mechanism underlying the development of metabolic dysfunction-associated steatotic liver disease (MASLD) and metabolic dysfunction-associated steatohepatitis (MASH). High glucose/high fat diet (HG/HFD) and excess alcohol consumption stimulate neutral lipid biosynthesis by synthesis of endogenous cholesterol and oxysterols, mainly 25-hydroxycholesterol (25HC). 25HC then penetrates the cell nuclei and activates DNA methyltransferase I (DNMT1), which, in turn, methylates CpG sites in promoter regions. This methylation process leads to the suppression of critical gene expressions associated with calcium-AMPK, insulin resistance, and phosphoinositide-3-kinase (PI3K)-AKT signaling pathways. Consequently, these events culminate in the accumulation of lipids, endoplasmic reticulum (ER) stress, lipotoxicity, inflammatory responses, mitochondrial depolarization, oxidative stress, and ultimately, cell death, the process from MASLD to MASH. Cholesterol lowering drug, HMGR inhibitor, statin lowers serum triglyceride via decreasing intracellular 25HC levels. ACS, acetyl-CoA synthetase; ADH, alcohol dehydrogenase.

There are two pathways of alcohol metabolism: *1*) most alcohol is metabolized by the alcohol dehydrogenase (ADH) catalyzed oxidation system; and *2*) microsomal ethanol oxidizing system (MEOS) in the liver (53). The ADH oxidation system metabolizes alcohol into acetaldehyde and acetic acid. MEOS becomes active if the ADH system cannot quickly remove ethanol by long-term heavy drinking. The core element of MEOS is cytochrome P450 family 2 subfamily E member 1 (CYP2E1), which requires oxygen for its catalytic process, producing free radicals that damage the liver. Excessive alcohol consumption increases the expression and activity of CYP2E1, activating carcinogens and hepatotoxins, and converting them into more toxic metabolites (54). DNA methylation of CYP2E1 plays an irreplaceable role in maintaining normal liver function (55). A similarity between HG-induced MASLD and AH remains unexplored. It is possible that alcohol is oxidized to acetaldehyde and further to acetate. The acetate can be degraded by the citric cycle to generate energy, ATP, via oxidation-phosphorylation. Excess acetate can be used to synthesize cholesterol and oxysterols such as 25HC, a potent endogenous epigenetic regulator. The alcohol-derived 25HC activates DNMT1 and subsequently silences key gene expressions involved in MAPK-ERK, calcium-AMPK, PI3K, and insulin signaling pathways, resulting in lipid accumulation, insulin resistance, and the development of AH as shown in Fig. 1. Therefore, exploring epigenetic modifiers holds promise as a potential approach for treating AH.

## GENERAL MECHANISM OF LARSUCOSTEROL ACTION

### Larsucosterol Downregulates Lipid Biosynthetic Pathways by Decreasing CpG Methylation in Promoter Regions of Key Genes Involved in Calcium-AMPK Signaling Pathway

Remarkably, in a model of HG-induced MASLD in human hepatocytes, larsucosterol exhibits a specific inhibition of DNMTs. This results in the conversion of ^5m^CpG to CpG in the promoter regions of 1,074 genes within hepatocytes, leading to the upregulation of genes associated with key signaling pathways such as MAPK-ERK, calcium-AMPK, and type II diabetes mellitus pathways (23). Demethylated signaling pathways regulate the gene expression of key elements and enzymes that decrease lipid biosynthesis and promote cell survival. The calcium, AMPK, and peroxisome proliferator-activated receptor (PPAR) signaling pathways are the master ones involved in the regulation of energy, lipids, and carbohydrate metabolisms. Peroxisome proliferator-activated receptor gamma coactivator 1-alpha (PGC-1α) is a key regulator of mitochondrial biogenesis, oxidative phosphorylation, and mitochondrial antioxidant defense, and it is also responsible for maintaining metabolic homeostasis (56). PGC-1α expression is upregulated by the cAMP responsive element binding protein (CREB) protein and the AMPK signaling pathway (57, 58). Larsucosterol upregulates expression of CREB and AMPK via demethylating ^5m^CpG in their promoter regions and subsequently increases intracellular PGC-1α levels. In addition, larsucosterol suppresses DNMT activities and demethylates ^5m^CpG in the key promoter regions. The demethylation upregulates calcium-AMPK signaling, resulting in inhibition of sterol regulatory element-binding transcription factor 1 (SREBP-1) activity, and further inhibits the expression of genes involved in lipids biosynthesis and transport pathways including 3-hydroxy-3-methylglutaryl-CoA reductase (HMGR), fatty acid synthase (FAS), acetyl-CoA carboxylase (ACC), and proprotein convertase subtilisin/kexin-type 9 (PCSK9) (24). The results strongly support the potential of larsucosterol in treating steatosis.

### Larsucosterol Downregulates Inflammatory Response through Decreasing the Promoter Methylation Levels of Key Genes

**Figure 2.**
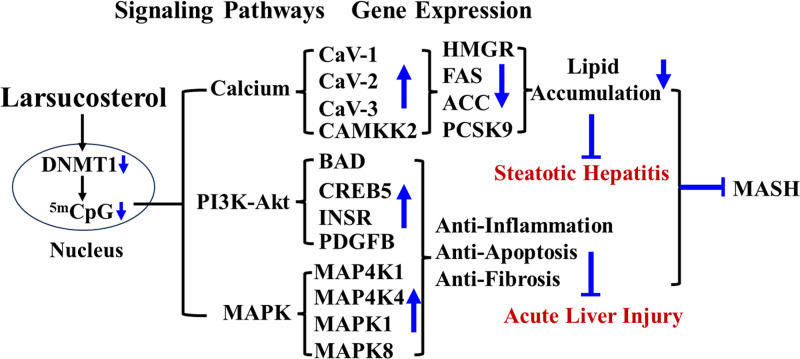
Schematic diagram depicting the general mechanism of larsucosterol for treating chronic and acute liver injury. Chronic liver diseases are currently believed to unfold in three primary stages: lipid accumulation, inflammation, and apoptosis. Larsucosterol at pharmaceutical concentrations enters nucleus, where inactivates DNA methyltransferase I (DNMT1), passively demethylates promoter ^5m^CpG and upregulate genes involved in MAPK, phosphoinositide-3-kinase (PI3K), calcium-AMPK signaling pathways, resulting in increases in their gene expressions and decreases lipid biosynthesis. RNA-Seq analysis showed that treatment with larsucosterol in hepatocytes significantly modulated gene expression in many clusters. The major affected clusters were genes involved in cholesterol and triglyceride metabolism, cell survival, and inflammation, which are regulated by MAPK, PI3K, and calcium signaling pathways. Genes associated with lipid biosynthesis were significantly downregulated, whereas genes associated with cell survival, antiapoptosis, and antioxidants were significantly upregulated. The errors represent upregulation or downregulation, the bar, block. MASH, metabolic dysfunction-associated steatohepatitis.

Previous reports have demonstrated that addition of larsucosterol suppresses inflammatory responses, by decreasing the expression of proinflammatory cytokines and decreasing nuclear factor kappa B subunit 1 (NF-κB) and tumor necrosis factor-alpha (TNFα) levels in the human THP-1 derived macrophages. Further analysis revealed that larsucosterol achieves this suppression by increasing PPARγ expression in the cells (32). The nuclear receptor peroxisome proliferator-activated receptors (PPARs) have been reported as important elements in regulating lipid metabolism and inflammatory responses in macrophages (59). Activation of PPARγ has been shown to reduce NF-κB nuclear translocation and inhibit NF-κB-dependent gene expressions (60). Multiple kinase pathways, including cAMP-dependent protein kinase (PKA), AMP-activated protein kinase (AMPK), and mitogen-activated protein kinase (MAPK), are implicated in the regulation of PPARγ gene expression and phosphorylation (61). Demethylation of ^5m^CpG in the promoter regions of AMPK and MAPK by larsucosterol upregulates their gene expressions and activates their signaling pathways, leading to increased PPARγ gene expression and its phosphorylation, which results in nuclear translocation. Recent studies have demonstrated that larsucosterol inhibits the activities of DNMTs and induces the demethylation of ^5m^CpG in the promoter regions of gene expressions associated with calcium-AMPK and MAPK signaling pathways. Therefore, it is reasonable to propose that larsucosterol suppresses inflammatory responses by demethylating ^5m^CpG in DNA promoter regions and activating the AMPK and MAPK signaling pathways (28). Moreover, the impact of both larsucosterol and the synthetic PPARγ agonist rosiglitazone on nuclear PPARγ levels was found to be similar (32). These findings suggest that larsucosterol regulates inflammatory responses through the PPARγ/IκB signaling pathway by inducing DNA ^5m^CpG demethylation in the promoter regions. These results provide evidence that larsucosterol not only decreases lipid biosynthesis but also suppresses inflammation and apoptosis via epigenomic modification. The multifunctionality of larsucosterol makes it a promising candidate for treating chronic and acute liver diseases as shown in Fig. 2.

## LARSUCOSTEROL MAY BE USEFUL IN TREATING CHRONIC LIVER DISEASES

MASLD and MASH are the most common chronic liver disease worldwide (62). In the transition from simple steatosis to MASH, inflammatory cytokines like TNFα, IL-1, and IL-6 exert a significant influence, with their concentrations directly correlating to the degree of fibrosis in MASH (63). Numerous studies have demonstrated that the larsucosterol administration not only reduces lipid accumulation and improves insulin resistance but also suppresses inflammation and enhances hepatocyte regeneration, both in vitro and in vivo (28, 31–37, 64).

In an established MASH mice model (33), larsucosterol was administered in an acute treatment regimen, involving two injections within a 14 h period, with mice subjected to overnight fasting before euthanasia. Results indicated that larsucosterol significantly decreased plasma triglyceride (TG), cholesterol (CHOL), and HDL-C levels. Liver function analysis, based on serum alanine aminotransferase (ALT) and aspartate aminotransferase (AST) levels, revealed no significant impact on liver function. These findings suggest that acute administration of larsucosterol significantly lowers circulating lipid levels in the MASLD mouse model. For long-term treatments on the established MASH mice model, mice received peritoneal injections once every 3 days for 6 wk while continuously being fed a high-fat diet (HFD). After the 6-wk injection period, mice were fasted for 5 h before euthanasia. Notably, the average liver weight significantly decreased in the larsucosterol-treated group. Furthermore, larsucosterol significantly reduced triglyceride, total cholesterol, free cholesterol, and free fatty acid levels. It also led to a significant reduction in serum alkaline phosphatase (ALK), ALT, and AST levels. These results suggest that larsucosterol treatment protects the liver from injury, possibly by suppressing hepatic inflammation.

**Figure 3.**
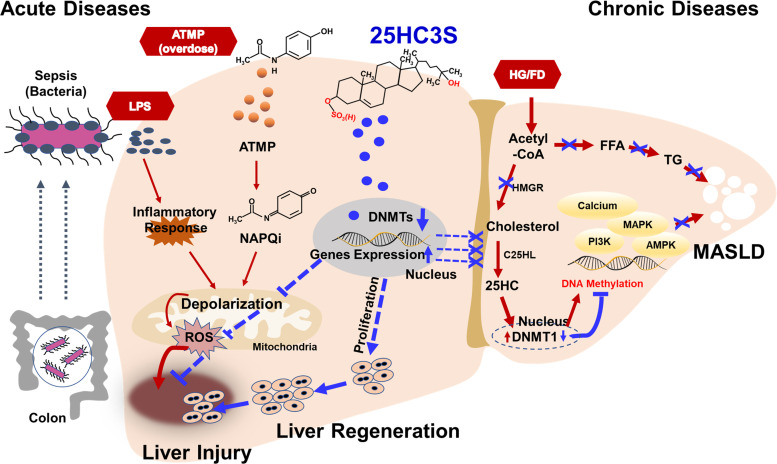
The potential therapeutic effects of larsucosterol, an endogenous epigenetic regulator, on chronic and acute liver diseases. Larsucosterol has been shown to improve lipid metabolism, inflammatory responses, and cell survival in liver diseases. In particular, it effectively treats nonalcoholic fatty liver disease induced by high glucose or high fat diets by reducing methylation of genes involved in lipid metabolism signaling pathways (Chronic Diseases). Moreover, larsucosterol promotes liver regeneration by decreasing DNA methylation, increasing the expression of genes involved in proliferation signaling pathways and subsequently keeping mitochondria polarized (Acute Diseases). For example, larsucosterol aids in the recovery of injured liver tissue caused by endotoxic lipopolysaccharides (LPS) and acetaminophen (ATMP) overdose by regulating relative genes to increase mitochondrial polarization and reduce reactive oxygen species (ROS) levels, resulting in recovery from liver injury. AMPK, AMP-activated protein kinase (AMPK) signaling pathway; C25HL, 25-hydroxycholesterol hydroxylase; DNMT1, DNA methyltransferase I; FD, high fat diet; FFA, free fatty acids; HG, high glucose; MAPK, Ras-Raf-MEK-ERK pathway; MASLD, metabolic dysfunction-associated steatotic liver disease; NAPQI, *N*-acetyl-*p*-benzoquinone imine; PI3K, phosphoinositide-3-kinase–protein kinase B/Akt (PI3K-PKB/Akt) pathway; TG, triglycerides.

Molecular-level analyses of both acute and long-term treatments with larsucosterol revealed substantial reductions in mRNA levels of genes implicated in lipid biosynthesis pathways, such as SREBP-1c, ACC, and FAS. Moreover, long-term treatments with larsucosterol resulted in a notable decrease in the expression of key enzymes associated with triglyceride synthesis, including glycerol-3-phosphate acyltransferase (GPAM), as well as lipid transfer proteins like microsomal triglyceride transfer protein and phospholipid transfer protein (PLTP). In addition, long-term treatments with larsucosterol downregulated the expression of genes involved in oxidized LDL uptake, namely, CD36, and scavenger receptor class B, member 1, both contributing to the development of foam cells, with CD36 also implicated in hepatic inflammation. These findings strongly indicate that dysregulation of lipid metabolism is closely associated with inflammatory conditions. Moreover, long-term treatments with larsucosterol significantly suppressed the mRNA expression of proinflammatory cytokines, including TNFα, interleukin 1α (IL-1α), and interleukin 1β (IL-1β). Taken together, these results underscore the considerable promise of larsucosterol as an innovative treatment for MASLD, MASH, and liver fibrosis, as illustrated in Fig. 3.

## LARSUCOSTEROL MAY BE USEFUL IN TREATING ACUTE LIVER DISEASES

Acute liver injury (ALI) and liver failure (ALF) represent life-threatening conditions characterized by profound hepatocyte injury, often accompanied by extensive necrosis and apoptosis within the liver (65). The most frequent causes of ALF are endotoxin LPS- and drug ATMP-induced hepatotoxicity and hepatitis (66, 67). Currently, there are no specific treatment available for other etiologies of ALF, and in severe cases, liver transplantation stands as the sole treatment option. Consequently, there is an urgent need for the development of more effective therapeutic strategies for ALF (68).

### Larsucosterol Alleviates LPS-Induced Acute Multiple Organ Injury in Mouse Models

Sepsis is a common complication following infection, shock, and severe trauma, stands as a major causes of mortality in patients in intensive care units (ICUs) (69). Upon pathogen infection, the host innate immune system is excessively activated; followed by the occurrence of a cytokine storm, which is characterized by high levels of proinflammatory cytokines and results in cell death and multiple organ dysfunction (70). Importantly, the liver is the main target of the inflammatory response, and it is one of the most severely affected organs in sepsis. ALI is recognized as an important cause of mortality in patients with sepsis (71). Unfortunately, no effective therapeutic approaches are currently available to cure this disease, and the pathophysiology of the disease is still not fully understood (72).

**Figure 4.**
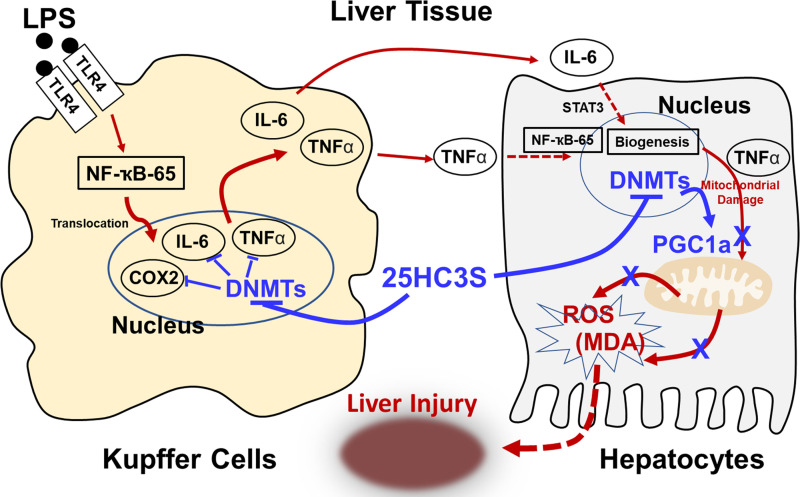
Schematic representation of the potential role of larsucosterol in the recovery of lipopolysaccharides (LPS)-induced acute liver injury. LPS-induced liver injury is characterized by the upregulation of proinflammatory cytokines, including cyclooxygenase (COX)-2, TNF-α, and IL-6, which damage hepatocyte mitochondria. Larsucosterol, an antagonist of DNA methyltransferase I (DNMTs), enters the nuclei and demethylates ^5m^CpG in promoter regions, resulting in the upregulation of PGC-1α gene expression. This leads to improved mitochondrial integrity and increased mitochondrial membrane polarization, which can promote recovery from acute liver injury.

The administration of larsucosterol has demonstrated efficacy in ameliorating liver, lung, and kidney function and reducing mortality in mouse models induced with LPS (31). Notably, when administered within 96 h of LPS-induced organ injury, the survival rate reached 90%, contrasting with a survival rate of 10% in animals not receiving larsucosterol. Time-course studies of apoptotic gene expression following LPS challenge in larsucosterol-treated mice were conducted to determine whether the larsucosterol-induced anti-inflammatory effects were preventive or therapeutic (31). The mRNA levels of genes involved in apoptosis were determined using the RT^2^ Profiler PCR Array. Cluster gram analysis showed that the profiles were very similar between control and treated groups at 3 and 6 h, but a significant difference was observed at 20 h. The profiles of the treated group at 20 h were more like the normal profile. The genes most significantly affected by larsucosterol were associated with immune system processes, autophagy processes, and apoptosis. These findings provide compelling evidence that larsucosterol exhibits therapeutic effects beyond prevention. Specifically, it demonstrates the ability to suppress inflammatory responses, inhibit apoptosis, and promote hepatocyte proliferation, as illustrated in Fig. 4 (30–32, 34).

### Larsucosterol Increases the ^5m^CpG Demethylation and Gene Expression Involved in Antiapoptosis and Proliferation Signaling Pathways and Keeps Mitochondrial Polarization in Mouse Models

In addition to sepsis-induced mortality, acute liver injury and liver failure caused by ATMP overdose is another significant clinical problem in most Western countries (73). Currently, the only clinically approved antidote is *N*-acetylcysteine (NAC), which promotes the recovery of hepatic GSH (74). If administered during the metabolism phase, GSH scavenges the reactive metabolite *N*-acetyl-*p*-benzoquinone imine (NAPQI). Recent studies have also demonstrated that NAC can also reconstitute mitochondrial GSH levels and scavenge reactive oxygen/peroxynitrite, thereby supporting mitochondrial bioenergetics. However, NAC has side effects and may not be efficacious following high overdoses (75).

Mitochondrial dysfunction, particularly the disruption of mitochondrial membrane potential (MMP), is considered the principal subcellular mechanism underlying hepatotoxicity induced by LPS and an overdose of ATMP (76, 77). This dysfunction leads to mitochondrial depolarization and leakage through the mitochondrial permeability transition (MPT), a crucial step in the processes of necrosis and apoptosis. MPT blockers, such as cyclosporine A, have the capability to prevent the loss of MMP and the initiation of cell death. There is a hypothesis suggesting that MPT plays a causal role in acute necrotic and apoptotic cell death (78). Moreover, the generation and release of reactive oxygen species (ROS) from compromised mitochondria play a crucial role in the pathogenesis of oxidative damage. They contribute to retrograde redox signaling from the mitochondria to the cytosol and nucleus. These ROS have the capacity to degrade polyunsaturated lipids, leading to the formation of malondialdehyde (MDA), a highly reactive compound that serves as a natural marker for oxidative stress (79). Therefore, mitochondrial dysfunction has been implicated in acute liver injury as well as many metabolic diseases, including MASLD and MASH (80). Maintaining mitochondrial polarization is the key point to effectively treat the acute liver/multiple organ injury and failure.

The administration of larsucosterol has been shown to promote the recovery of liver function in LPS- and ATMP-induced ALF mouse models (30, 31). Larsucosterol significantly decreases serum LDH, ALT, and AST and redeems most indicators of liver function to normal levels within 48 h, as well as significantly decreasing mortality in both models. Additional investigations have revealed that larsucosterol is effective in ameliorating acute multiple organ injuries induced by LPS and ATMP, particularly in the lung, kidney, and liver. This beneficial effect is attributed to the stabilization and polarization of mitochondria, along with a reduction in intracellular oxidants, ultimately leading to a decrease in mortality (30, 31). In a previous study, ATMP treatment resulted in a 30% reduction in MMP compared with normal human hepatocytes. Conversely, both propofol glyceryl ether (PG) and larsucosterol + PG demonstrated dose-dependent reductions in MMP loss, with larsucosertol + PG exhibiting superior protective effects. Examination of the impact of larsucosterol on mitochondrial polarization revealed that ATMP overdose significantly increased ROS levels by 40% and MDA levels by 30-fold. However, treatment with larsucosterol restored both ROS and MDA levels to normal, whereas slightly elevating glutathione (GSH) levels (30). These findings indicate that larsucosterol mitigates ROS and MDA levels by preserving mitochondrial membrane polarization and the integrity of this critical cellular organelle, suggesting its potential as a therapeutic agent for preventing mitochondrial dysfunction-induced cellular damage and death.

Previous studies have indicated that larsucosterol facilitates the recovery of hepatic injuries by inducing DNA ^5m^CpG demethylation in the promoter regions of genes associated with crucial PI3K-Akt and MAPK signaling pathways (30). These findings illustrate the mechanism by which larsucosterol regulates vital cell signaling pathways through epigenetic regulation, serving as means to prevent both acute organ injury and chronic liver diseases, as depicted in Figs. 2 and 3.

Further studies have shown that larsucosterol increases the ^5m^CpG demethylation and gene expression involved in antiapoptosis and proliferation signaling pathways in mouse models, indicating that the compound may promote recovery in ALF by facilitating hepatocyte proliferation and inhibiting apoptosis via epigenomic modification (30). The experiments using ATMP- and LPS-induced mouse models have demonstrated that larsucosterol promotes the recovery of injured liver function and decreases mortalities due to LPS or ATMP overdose. One exciting result showed that while intravenous (iv) injection of LPS (5 mg/kg body wt) alone is lethal to mice, co-injection of larsucosterol (1.5 mg/kg body wt) with LPS can reduce the mortality rate to 20% within 24 h. These findings suggest that larsucosterol has the potential to serve as a biomedicine for treatment of ALF (31).

### Larsucosterol Promote Liver Regeneration in Hepatectomy Animal Models

Hepatocyte regeneration plays a pivotal role as an adaptive response in both chronic and acute liver diseases (81). This process is crucial for maintaining adequate liver function, particularly following hepatocyte destruction induced by factors such as lipid accumulation, inflammation, and other injuries (82). Although the factors regulating hepatocyte regeneration are not fully understood, it is believed that growth factors and cytokines from both extrahepatic sites and the liver itself contribute to this intricate process. Quiescent adult hepatocytes possess the ability to replicate in response to physiological stimuli, and promoting their proliferation is essential for successful regeneration and recovery from liver injury. In a mouse hepatectomy model (64), larsucosterol upregulates the expression of genes associated with cell replication, including Wt1 (Wilms' tumor 1), PCNA (proliferating cell nuclear antigen), cMyc (myelocytomatosis oncogene), cyclin A, FoxM1b (Forkhead Box M1b), and CDC25b (M-phase inducer phosphatase 2), whereas concurrently downregulating the expression of cell cycle arrest gene Chek2 (checkpoint kinase 2) and the apoptotic gene Apaf1 (apoptotic peptidase activating factor 1). In addition, larsucosterol induces hepatic DNA replication and the PCNA labeling index, signifying its capacity to enhance hepatocyte proliferation. Recent studies showed that larsucosterol greatly demethylated ^5m^CpG in promoter regions of DUSP (dual-specificity phosphatase) genes, including DUSP8, DUSP1, and DUSP7, and their downstream genes, CREB5, peroxiredoxin 6, BCL2-associated agonist of cell death, and ERK (extracellular signal-regulated kinases), and increased their expression (28). The transcribed proteins from these genes are responsible for cell survival and proliferation. Therefore, the effects of larsucosterol on promoting cell survival/proliferation and alleviating oxidative stress occur through inhibiting DNMTs and increasing the expression of the DUSP family, especially DUSP8 and their downstream elements. The outcomes of these studies suggest that larsucosterol has the potential to contribute to the recovery of injured organs, particularly in the context of acute organ failure, by fostering cell regeneration.

## CLINICAL TRIALS

### Larsucosterol May Serve as a Potential Novel Biomedicine for Treatment of Chronic and Acute Liver Diseases

Both in vitro and in vivo data have shown that larsucosterol has strong potential to treat chronic and acute liver diseases (30, 31, 33, 64). Preclinical trial data have shown that larsucosterol is highly conserved across all seven mammals studied to date (83). Clinical trials using larsucosterol for safety and efficacy tests have been conducted in both normal humans and patients with chronic and acute liver diseases. Multiple clinical trials have shown that larsucosterol is well tolerated in more than 350 subjects at all doses tested via oral, intramuscular, and intravascular administration (83–85). The promising data from *phase 1b* and *phase 2a* indicate that larsucosterol seems to be an innovative and potentially efficacious new therapy for the treatment of MASH (85) and AH (27).

### Safety and Efficacy Tests in Healthy Subjects and in Patients with MASLD and MASH (*Phase 1b*)

As a potent epigenetic regulator, larsucosterol has strong potential as a novel biomedicine for the treatment of MASLD and MASH. Recent reports have shown that daily oral administration of DUR-928 for 4 wk in subjects with MASH resulted in an overall improvement in liver enzymes, decreasing ALT and AST by 35%; liver fat content decreased by 20% using noninvasive, quantitative assessment of liver fat, MRI-proton density fat fraction (MRI-PDFF) as an endpoint in MASH trials; serum lipid profiles, decreasing LDL-cholesterol by 12%, and certain biomarkers (85). Additional data on efficacy signals have shown an overall improvement in liver stiffness measured by either magnetic resonance or transient elastography. The liver fibrosis marker, pro-C3, as well as insulin resistance assessed by HOMA-IR (homeostatic model assessment for insulin resistance) also showed improvement. These results suggest that epigenetic regulation of gene expression and their signaling pathways is an attractive approach to treating MASH (85).

### Safety and Efficacy Tests in Patients with AH (*Phase 2a*)

A *phase 2a* clinical trial of larsucosterol for AH (Clinical Trials Identifier: NCT03432260) yielded encouraging results (27). Currently, there are no FDA-approved therapies for AH (50). A meta-analysis of 77 studies published between 1971 and 2016, including data from 8,184 patients, revealed that the overall mortality rate from AH was 26% at 28 days, 29% at 90 days, and 44% at 180 days (86). Compared with the meta-analysis results, *phase 2a* clinical trials showed that larsucosterol significantly increased survival rates (27). In the 28-day follow-up period of the *phase 2a* clinical trial, 100% of patients (*n* = 19), including 12 patients with severe AH, survived, compared with a historical 28-day mortality rate of 26%. Significant reductions from baseline in serum total bilirubin levels were observed at both *day 7* and *day 28* after larsucosterol dosing, including in patients with moderate AH at *day 7* and in patients with severe AH at *day 28*. Fourteen (74%) of all subjects including eight (67%) of subjects with severe AH were discharged in less than 72 h after receiving a single infusion. In addition, reductions in end-stage liver disease (MELD) scores from baseline were observed at *day 7* and *day 28*, with patients with moderate AH having statistically significantly lower MELD scores at *day 28*. All eight patients with severe AH in the 30- or 90-mg dose cohorts were treatment responders, with their Lille scores being statistically lower than those of well-matched patients from the Observational Arm and Study-Steroid Arm of the DASH Consortium trial (*P* < 0.01). Both AST and ALT enzymes decreased rapidly in patients with severe AH in the 30- or 90-mg dose cohorts, with ALT being statistically significantly lower than those in the DASH patients in a cross-study comparison (27).

### Safety and Efficacy Tests in Patients with AH (*Phase 2b*)

The US FDA granted larsucosterol Fast Track Designation for the treatment of AH in 2020, and larsucosterol is currently being evaluated in a worldwide *phase 2b* clinical trial (NCT04563026). This trial is a randomized, double-blind, placebo-controlled, *phase 2b* clinical trial evaluating safety and efficacy of larsucosterol (an experimental medication) in patients with AH. It has been claimed that the enrollment of 307 patients has been completed. Larsucosterol was well-tolerated; both dose groups had numerically fewer adverse events than the standard of care. Reduction in 90-day mortality is viewed as an advancement, as steroids do not show an effect on mortality past 28 days. The company is carefully analyzing the results (87).

## PERSPECTIVE

Chronic metabolic syndrome, including obesity, hyperlipidemia, atherosclerosis, diabetes, MASLD, and MASH, is closely associated with cardiovascular diseases (CVD), a leading cause of human deaths worldwide (88). These diseases are caused by systematic disorders of metabolic regulation, which are difficult to be perfectively treated with one agonist or one antagonist (89). However, the majority of therapies developed to treat metabolic syndrome target specific enzymes, receptors, or transporters (90). These treatments form complexes with their targets, which can accumulate in the cells and lead to side effects. A potentially more effective treatment for metabolic syndrome could involve a systematic regulator at the transcriptional level, such as larsucosterol. This compound has the capability to epigenetically regulate the transcription of proteins and enzymes involved in pathophysiological processes. Current data confirm that larsucosterol globally regulates the gene expression involved in inflammatory response, lipid metabolism, and apoptosis, which are the major pathogenesis occurring in the disease development.

The potential of larsucosterol extends to the management of precipitating factors in acute-on-chronic liver injury (ACLI). ACLI is a syndrome separate from the natural progression of liver diseases, often triggered by infections (91–94). Through its demonstrated ability to suppress LPS-induced inflammatory responses and mitigate ATMP-induced liver injury via calcium-AMPK and MAPK-ERK signaling through epigenetic regulation, larsucosterol emerges as a promising novel biomedicine for the treatment of both chronic and acute organ injuries, including liver diseases.

The progression of acute organ injury (AOI) can culminate in acute organ failure (AOF), encompassing conditions such as acute liver, lung, and kidney failure, which can lead to the sudden demise of previously healthy individuals (95). Most cases of AOF are attributed to endotoxin-induced liver injury (65). The pathophysiology of endotoxic shock involves four distinct stages, and early administration of larsucosterol has already demonstrated significant improvements in organ function and a reduction in mortality in LPS-induced animal models, as well as in clinical *phase 1* and *2a* trials. Hence, larsucosterol holds the potential to be an effective therapeutic option, particularly in the early stages of septic shock.

Larsucosterol has been tested in animals and humans. Although additional testing is needed, current data suggest that larsucosterol has the potential to promote recovery from metabolic syndromes and may prevent and/or treat AOI and AOF. Clinical trial data from treatments of patients with MASH and AH support the potential of larsucosterol as a novel therapeutic option.

## GRANTS

This study was supported by a VA Merit Review grant, US Department of Veterans Affairs under Grant No. BX003656 and DURECT Corporation, Research Agreement.

## DISCLOSURES

S.R. and Virginia Commonwealth University obtain license-related payments from DURECT Corporation, Cupertino, CA. None of the other authors has any conflicts of interest, financial or otherwise, to disclose.

## AUTHOR CONTRIBUTIONS

Y.W. analyzed data; Y.W. and S.R. prepared figures; Y.W. and S.R. drafted manuscript; J.R. and S.R. edited and revised manuscript; S.R. approved final version of manuscript.
